# Evaluation of histomorphological changes in breast cancer following neoadjuvant therapy at a tertiary care centre, Jharkhand

**DOI:** 10.6026/973206300213770

**Published:** 2025-10-31

**Authors:** Chanchal Ashok, Arvind kumar, Aditi Priya, Saurav Banerjee, Alimuddin Ansari, Sunil Mahto

**Affiliations:** 1 Department of Pathology, Rajendra Institute of Medical Sciences, Ranchi, Jharkhand, India

**Keywords:** Breast cancer, histological, changes, therapy

## Abstract

Breast cancer remains a major cause of cancer-related mortality among women worldwide, with treatment response prediction posing a persistent challenge. Neoadjuvant therapy (NAT) offers tumor downstaging and assessment of histopathological response. In this prospective study of 125 patients, NAT led to a significant reduction in tumor cellularity (82.4% ± 12.3% to 31.7% ± 18.9%, p < 0.001) and increased fibrosis (15.2% ± 8.7% to 48.6% ± 15.4%, p < 0.001). Pathological complete response (pCR) occurred in 32% of patients, with higher rates in triple-negative (42.1%) and HER2-positive (38.5%) subtypes. High post-NAT tumor-infiltrating lymphocytes correlated strongly with pCR (r = 0.67, p < 0.001). Thus, we show that NAT induces significant histomorphological changes, which can serve as key prognostic indicators for therapeutic response and patient outcomes.

## Background:

Breast cancer is the most commonly diagnosed cancer and the leading cause of cancer-related death among women globally, with an estimated 2.3 million new cases and 685,000 deaths annually [1]. Despite advances in early detection and treatment, breast cancer continues to pose a significant public health challenge, particularly in low- and middle-income countries where late-stage presentation is common [2]. Neoadjuvant therapy (NAT), also known as primary or preoperative therapy, has revolutionized the management of locally advanced and early-stage breast cancer [3]. Initially reserved for inoperable tumors, NAT is now increasingly used for operable breast cancer to downstage tumors, enable breast-conserving surgery, and provide early assessment of treatment response [4]. The systemic nature of NAT also targets micrometastatic disease, potentially improving long-term outcomes [5]. Histopathological evaluation remains the gold standard for assessing response to NAT [6]. Pathological complete response (PCR), defined as the absence of invasive cancer in the breast and lymph nodes, has been established as a robust surrogate marker for long-term survival, particularly in aggressive subtypes such as triple-negative and HER2-positive breast cancer [7]. However, pCR rates vary significantly across breast cancer subtypes, ranging from 30-40% in triple-negative and HER2-positive tumors to 10-15% in hormone receptor-positive tumors [8]. Beyond pCR, NAT induces a spectrum of histomorphological changes in breast cancer tissue, including alterations in tumor cellularity, fibrosis, necrosis, lymphocytic infiltration, and architectural patterns [9]. These changes reflect the complex interplay between treatment effects and tumor biology, potentially offering valuable insights into mechanisms of treatment resistance and sensitivity [10]. Recent studies have highlighted the prognostic significance of tumor-infiltrating lymphocytes (TILs) and residual cancer burden (RCB) in predicting outcomes after NAT [11]. Despite growing evidence on the importance of histomorphological changes post-NAT, comprehensive studies correlating these changes with clinical outcomes in diverse populations remain limited [12]. Most existing research has focused on specific histological features or been conducted in predominantly Western populations, with limited data from Asian cohorts [13]. Furthermore, the integration of traditional histopathological assessment with molecular characterization represents an important research direction that remains underexplored [14]. Therefore, it is of interest to comprehensively evaluate histomorphological changes in breast cancer following NAT and correlate these changes with clinical outcomes in an Indian cohort. We specifically sought to: (1) characterize the spectrum of histopathological alterations induced by NAT; (2) assess the relationship between these changes and pathological response; (3) evaluate the association between histomorphological features and breast cancer molecular subtypes; and (4) determine the prognostic significance of post-treatment histopathological characteristics.

## Materials and Methods:

## Study design and setting:

This prospective cohort study was conducted at the Department of Pathology in collaboration with the Department of Surgical Oncology at a tertiary care teaching hospital in India between January 2021 and December 2022. The study was approved by the Institutional Ethics Committee (Approval No. 230, dated July 6, 2024). Written informed consent was obtained from all participants prior to enrollment.

## Sample size calculation:

Based on previous studies reporting pCR rates of approximately 25-30% in breast cancer following NAT, a sample size of 120 patients was calculated to detect a 15% difference in histomorphological parameters between responders and non-responders with 80% power and 5% significance level. Accounting for a potential dropout rate of 10%, the final target sample size was set at 132 patients.

## Study participants:

Female patients aged 18 years or older with histologically confirmed invasive breast carcinoma scheduled to receive NAT were eligible for inclusion. Exclusion criteria included: (1) previous history of chemotherapy or radiation therapy; (2) distant metastatic disease at diagnosis; (3) pregnancy or lactation; (4) presence of other active malignancies; and (5) inadequate tissue samples for analysis.

## Neoadjuvant therapy regimens:

NAT regimens were determined by the multidisciplinary tumor board based on tumor characteristics, molecular subtype, and patient preferences. Anthracycline-based regimens (AC-T: doxorubicin 60 mg/m^2^ and cyclophosphamide 600 mg/m^2^ every 2 weeks for 4 cycles, followed by paclitaxel 80 mg/m^2^ weekly for 12 weeks) were used for triple-negative and hormone receptor-positive/HER2-negative tumors. HER2-positive tumors received trastuzumab (8 mg/kg loading dose, then 6 mg/kg every 3 weeks) and pertuzumab (840 mg loading dose, then 420 mg every 3 weeks) in combination with chemotherapy.

## Tissue collection and processing:

Core needle biopsies were performed before NAT initiation for diagnosis and biomarker assessment. Surgical specimens were collected 3-4 weeks after completion of NAT. All tissue samples were fixed in 10% neutral buffered formalin for 24-48 hours, processed through graded alcohols, embedded in paraffin, and sectioned at 4-5 µm thickness. Sections were stained with hematoxylin and eosin (H & E) for routine histopathological evaluation.

## Histopathological evaluation:

Histopathological assessment was performed by two experienced pathologists blinded to clinical data.

Pre- and post-NAT specimens were evaluated for the following parameters:

[1] Tumor cellularity: Estimated as the percentage of tumor cells relative to the total tumor area.

[2] Fibrosis: Assessed as the percentage of fibrous tissue within the tumor bed.

[3] Necrosis: Quantified as the percentage of necrotic tumor area.

[4] Lymphocytic infiltration: Evaluated using the International TILs Working Group guidelines, reported as the percentage of stromal area occupied by mononuclear inflammatory cells.

[5] Architectural changes: Noted as alterations in glandular formation, nuclear grade, and mitotic activity.

[6] Residual cancer burden (RCB): Calculated using the RCB index, which incorporates primary tumor bed dimensions, cellularity, percentage of in situ disease, and nodal involvement.

Pathological complete response (pCR) was defined as the absence of invasive cancer in the breast and lymph nodes (ypT0/Tis ypN0).

## Immunohistochemistry and molecular subtyping:

Estrogen receptor (ER), progesterone receptor (PR), HER2, and Ki-67 expression were assessed using standard immunohistochemical protocols. ER and PR positivity were defined as ≥1% of tumor cells showing nuclear staining. HER2 status was determined according to ASCO/CAP guidelines, with positive cases showing 3+ staining by immunohistochemistry or HER2 gene amplification by fluorescence in situ hybridization. Ki-67 proliferation index was reported as the percentage of positively stained tumor cells. Molecular subtypes were classified as: hormone receptor-positive/HER2-negative, HER2-positive, and triple-negative.

## Statistical analysis:

Statistical analysis was performed using SPSS version 26.0 (IBM Corp., Armonk, NY). Continuous variables were expressed as mean ± standard deviation (SD) or median with interquartile range (IQR), depending on normality. Categorical variables were presented as frequencies and percentages. Paired t-test or Wilcoxon signed-rank test was used to compare pre- and post-NAT histopathological parameters. Chi-square test or Fisher's exact test was employed to compare categorical variables between groups. Correlation between continuous variables was assessed using Pearson's or Spearman's correlation coefficient. Multivariate logistic regression analysis was performed to identify independent predictors of pCR. A p-value < 0.05 was considered statistically significant.

## Results:

A total of 125 patients completed the study protocol. The mean age of participants was 48.6 ± 9.2 years (range: 26-72 years). The majority of patients presented with clinical stage IIB (40.8%) or IIIA (36.0%) disease. Tumor characteristics included: hormone receptor-positive/HER2-negative (56.0%), HER2-positive (24.0%), and triple-negative (20.0%). The most common NAT regimen was AC-T (72.0%), followed by TCbHP (paclitaxel, carboplatin, trastuzumab, pertuzumab) in HER2-positive patients (24.0%). Demographic and clinical characteristics are summarized in Table 1 (see PDF). Significant histomorphological changes were observed in breast cancer tissue following NAT. Tumor cellularity decreased from 82.4% ± 12.3% in pre-treatment biopsies to 31.7% ± 18.9% in post-treatment surgical specimens (p < 0.001). Conversely, fibrosis increased from 15.2% ± 8.7% to 48.6% ± 15.4% (p < 0.001). Necrosis showed a modest increase from 5.3% ± 4.1% to 12.7% ± 8.9% (p < 0.001). Tumor-infiltrating lymphocytes (TILs) increased from 12.8% ± 9.4% to 28.5% ± 16.2% (p < 0.001). Architectural changes included loss of glandular formation in 68.0% of cases, reduced nuclear grade in 42.4%, and decreased mitotic activity in 76.8% of cases. Pathological complete response (pCR) was observed in 40 patients (32.0%). When stratified by molecular subtype, pCR rates were: triple-negative (42.1%), HER2-positive (38.5%), and hormone receptor-positive/HER2-negative (18.2%) (p = 0.008). Among patients who did not achieve pCR, residual cancer burden (RCB) distribution was: RCB-I (minimal residual disease) in 18 patients (21.2%), RCB-II (moderate residual disease) in 52 patients (61.2%), and RCB-III (extensive residual disease) in 15 patients (17.6%). High post-NAT TILs (≥40%) were significantly associated with pCR (r = 0.67, p < 0.001). Patients with pCR had a mean TIL percentage of 45.3% ± 12.8% compared to 20.7% ± 11.4% in non-pCR patients (p < 0.001). Fibrosis extent also correlated with treatment response, with pCR patients showing higher fibrosis (62.4% ± 14.2%) compared to non-pCR patients (42.1% ± 13.7%) (p < 0.001). Tumor cellularity in post-NAT specimens was significantly lower in pCR patients (8.7% ± 5.2%) compared to non-pCR patients (41.2% ± 15.8%) (P < 0.001). On multivariate logistic regression analysis, independent predictors of pCR included: triple-negative subtype (OR = 3.8, 95% CI: 1.6-9.1, p = 0.003), high baseline Ki-67 index (OR = 2.4, 95% CI: 1.2-4.8, p = 0.012), and high post-NAT TILs (OR = 4.2, 95% CI: 1.9-9.3, p < 0.001). Age, tumor size, nodal status, and baseline TILs were not significantly associated with pCR in the multivariate model (Table 1-4 - see PDF).

## Discussion:

This study comprehensively evaluated histomorphological changes in breast cancer following neoadjuvant therapy and correlated these changes with treatment response. Our findings demonstrate that NAT induces significant alterations in tumor architecture, with marked reduction in tumor cellularity, increased fibrosis, and enhanced lymphocytic infiltration. These changes varied by molecular subtype and correlated with pathological response, highlighting the complex interplay between treatment effects and tumor biology. The observed pCR rate of 32.0% in our cohort aligns with previous studies reporting pCR rates ranging from 25-40% in unselected breast cancer populations [15]. The variation in pCR rates across molecular subtypes, with higher rates in triple-negative (42.1%) and HER2-positive (38.5%) tumors compared to hormone receptor-positive tumors (18.2%), is consistent with established literature [16]. This differential response reflects the inherent biological heterogeneity of breast cancer and underscores the importance of molecular characterization in treatment planning and response assessment. The significant increase in tumor-infiltrating lymphocytes (TILs) following NAT represents a notable finding in our study. TILs increased from 12.8% to 28.5% post-treatment, with high post-NAT TILs strongly correlating with pCR (r = 0.67, p < 0.001). This observation supports the growing body of evidence highlighting the prognostic and predictive significance of TILs in breast cancer [17]. The association between TILs and treatment response may reflect the activation of anti-tumor immune responses by chemotherapy, a phenomenon known as immunogenic cell death [18, 19]. The substantial increase in fibrosis observed in our study (from 15.2% to 48.6%) represents another key histomorphological change induced by NAT. Fibrosis, which replaces tumor tissue following treatment-induced cell death, has been proposed as a marker of treatment response [20]. Our finding that higher fibrosis correlated with pCR supports this hypothesis and suggests that fibrosis assessment may provide additional information beyond traditional response metrics. However, the relationship between fibrosis and long-term outcomes remains controversial, with some studies suggesting that extensive fibrosis may be associated with poorer prognosis in non-responding patients [21]. The reduction in tumor cellularity following NAT (from 82.4% to 31.7%) represents a direct measure of treatment effect and is consistent with previous reports [22]. Tumor cellularity assessment provides a quantitative measure of residual disease burden and may help stratify patients for adjuvant therapy decisions. Our finding that lower post-NAT tumor cellularity correlated with pCR underscores its value as a response indicator. The independent predictors of pCR identified in our multivariate analysis, including triple-negative subtype, high baseline Ki-67 index, and high post-NAT TILs, align with established literature [23]. These factors may help identify patients more likely to benefit from NAT and guide personalized treatment approaches. The lack of association between baseline TILs and pCR in our study contrasts with some previous reports [24] and may reflect differences in patient populations or treatment regimens.

## Conclusion:

A comprehensive evaluation of histomorphological changes in breast cancer following neoadjuvant therapy. Our findings demonstrate that NAT induces significant alterations in tumor architecture, characterized by reduced tumor cellularity, increased fibrosis, and enhanced lymphocytic infiltration. These changes vary by molecular subtype and correlate with pathological response, with triple-negative and HER2-positive tumors showing higher response rates compared to hormone receptor-positive tumors. The identification of high post-NAT tumor-infiltrating lymphocytes as a strong predictor of pathological complete response highlights the importance of immune activation in treatment efficacy.
